# Supplementary material for: A Meiotic Drive Element in the Maize Pathogen Fusarium verticillioides Is Located Within a 102 kb Region of Chromosome V
Source: G3 (Bethesda). 2016 Jun 10;6(8):2543–52. doi: 10.1534/g3.116.029728 (PMC4978907; doi:10.1534/g3.116.029728)
Supplement: Supplemental Material [file supp_6_8_2543__index.html]

A Meiotic Drive Element in the Maize Pathogen Fusarium verticillioides Is Located Within a 102 kb Region of Chromosome V — Supplemental Material 

# A Meiotic Drive Element in the Maize Pathogen *Fusarium verticillioides* Is Located Within a 102 kb Region of Chromosome V

## Supplemental Material for Pyle *et al.*, 2016

**Files in this Data Supplement:**

- Figure S1 - Skc1 homologs exist within the *F. oxysporum* species complex. (.pdf, 194 KB)
- Figure S2 - A Clustal W alignment of the *FVEG\_03165-FVEG\_03164* intergenic region from Fv999-*SkK* and Fv149-*SkS*. (.pdf, 602 KB)
- Figure S3 - Sequence alignment of putative Fveg\_15999 homologs. (.pdf, 191 KB)
- Figure S4 - Sequence alignment of putative Fveg\_03194 homologs. (.pdf, 215 KB)
- Figure S5 - Sequence alignment of putative Fveg\_03197 homologs. (.pdf, 222 KB)
- Table S1 - Oligonucleotide primers for CAPS markers. (.pdf, 168 KB)
- Table S2 - CAPS marker sizes. (.pdf, 87 KB)
- Table S3 - Genotypes of the Fv999 (*SKK*) × Fv149 (*SKS*) mapping population at each CAPS location. (.pdf, 75 KB)
