# Supplementary material for: A Meiotic Drive Element in the Maize Pathogen Fusarium verticillioides Is Located Within a 102 kb Region of Chromosome V
Source: G3 (Bethesda). 2016 Jun 10;6(8):2543–52. doi: 10.1534/g3.116.029728 (PMC4978907; doi:10.1534/g3.116.029728)
Supplement: Supplemental Material [file supp_g3.116.029728_FigureS1.pdf]

```
                             10        20        30        40        50
                    ....|....|....|....|....|....|....|....|....|....|
Fv Skc1             MSTNSDTM-PGVILHLSSDDLDVLLDKKL----QP-IYTMLA-------T 37
Fo ENH69922.1        MSTNSDTM-PGITLHLSADDLERLLDRKL----QP-IFTKLAAIEQDLAT 44
Fo ENH68412.1        MSTNSDAM-PGITLHLSAADLEQLFDRKLKEVL----DEALQPIHSKLDA 45
Fo EMT68528.1        MSTNSNTM-PGIILHLSADDLEQLFDRKLKEVLDEVLDEALQPIHSKLDA 49
Fo EXL53286.1        MSTNSDAM-PGITLHLSAADLEQLFDRKLNEALQP-IYSKLDAIEEKLDA 48
Fo EXK45277.1        MSTNSDAM-PGITLHLSAADLEQLFDRKLNEALQP-IYSKLDAIEEKLDA 48
Fo KNB05864.1        MSTNSGPIGPGITLHLSAADLEQLFDRKLNEALQP-IYSKLDAIEEKLDA 49
Fo EWZ35922.1        MPG-------IIIFHASRDDLERIFDEKL----QP--------IKDKLDA 31
Fo EXA38056.1        MPG-------IIIFHASKDDLERIFDEKL----QP--------IKDKLDA 31

                             60        70        80        90
                    ....|....|....|....|....|....|....|....|.
Fv Skc1             IIKKLDDLTDRVEAVEGLAKKIPDMEVDIHSQD-------- 70
Fo ENH69922.1        IKADLKNLTARVGVVEGLAKKIPNMEVDLHSQD-------- 77
Fo ENH68412.1        IEEKLEKLTARVGVVEGLTAKIPNMEDDLRSQE-------- 78
Fo EMT68528.1        IEEKLEKLTARVEVVEGLAAKIPNMEDDLRSQD-------- 82
Fo EXL53286.1        IEEKFEKLTARVGVVEGLAAKIPNMEDDLRSQD-------- 81
Fo EXK45277.1        IEEKLEKLTARVGVVEGLTAKIPNMEDDLRSQD-------- 81
Fo KNB05864.1        IEEKLEKLTARVGVVEGLTAKIPNMEDDLRSQD-------- 82
Fo EWZ35922.1        INKMLDRLTN-----HGLAPKTPPIETDIRSRDYNLMPSQA 67
Fo EXA38056.1        INKMLDRLTN-----HGLAPKTPPIETDIRSRD-------- 59
```

Figure S1 Skc1 homologs exist within the *F. oxysporum* species complex. A BLASTP search of NCBI's non-redundant protein database with *F. verticillioides* Skc1 returned eight putative homologs, all from *F. oxysporum* species. The sequences were imported into Bioedit 7.2.5 and aligned with Clustal W. Columns were shaded according to the BLOSUM62 similarity matrix. Each sequence is identified by its accession number: Skc1, *F. verticillioides*; ENH69922.1, *F. oxysporum* f. sp. *cubense* race 1; EMT68528.1, *F. oxysporum* f. sp. *cubense* race 4; EXL53286.1, *F. oxysporum* f. sp. *radicis-lycopersici;* ENH68412.1, *F. oxysporum* f. sp. *cubense* race 1; EXK45277.1, *F. oxysporum* f. sp. *melonis*; KNB05864.1, *F. oxysporum* f. sp. *lycopersici*; EWZ35922.1, *F. oxysporum* Fo47; EXA38056.1, *F. oxysporum* f. sp. *pisi.* Only 82 amino acids from the C terminal end of KNB05864.1 were used in the analysis.
