# Supplementary material for: A Meiotic Drive Element in the Maize Pathogen Fusarium verticillioides Is Located Within a 102 kb Region of Chromosome V
Source: G3 (Bethesda). 2016 Jun 10;6(8):2543–52. doi: 10.1534/g3.116.029728 (PMC4978907; doi:10.1534/g3.116.029728)
Supplement: Supplemental Material [file supp_g3.116.029728_FigureS2.pdf]

```
          FVEG_03165
Fv999  GCGAATGACCTGGGGAGCCATAATCGCCCATCCGCGTCGATTTGCAGTGGTACTTGGTAGTGAGGTCAAG 70
Fv149  GCGAATGACCTGGGGAGCCATAATCGCGC----------------------------------------- 29

Fv999  AGTGCAAGTGGGCCTTAAATCGCTCATGCCTTATTTGGGTTTCCGCAACTCAACTTAGCTTCAACAAGCT 140
Fv149  ---------------------GCTTCTTCCTTTTTCGGG------------------------------- 47

Fv999  TTAATGGAGGAAGGTTTAACTCAAGAGTTCGCTTTTATAAACCAGGCGTCCATCTTCTGTCTTCTGTTTA 210
Fv149  ------------GGCTTGTCCGTAGACT-----------AATCGGGCGG--------------------- 73

Fv999  CTTTCTTCCTTACACACAATTCCATCAATCCACTACACAATTCCATCAATCCACTACACAATTCCATCAA 280
Fv149  ---------------------------------------------------------------------- 73

Fv999  TCCACTACACAAACACAAGTTGTCTATCAAGAAATCATCTCTCATTCTATCTCCCAGATCACCAATCAAT 350
Fv149  ---------------------------------------------------------------------- 73

Fv999  ACAGCCATGTCTACCAACTCAGACACCATGCCTGGCGTTATCCTCCACCTTTCATCAGATGATCTTGATG 420
Fv149  ---------------------------GTGGTGGGCGCTGT-------------------------AGTG 91

Fv999  TTTTGCTTGATAAGAAGCTACAGCCTATTTACACTATGCTGGCTACTATTATCAAGAAACTTGATGATCT 490
Fv149  TTGCGCTTGCGGGGGATCG------------------GCTGGAGAC-GGGAGGGGAAAAGTTGA----CT 138

Fv999  TACCGACCGTGTTGAGGCAGTTGAAGGCCTGGCGAAAAAGATCCCGGACATGGAAGTCGATATCCATTCT 560
Fv149  TAT-----GT-TTGACTTGGTTTTAGGTC-GGTGCTAAAGCTCCTGGA----GAAG-------------- 183

Fv999  CAAGACTAGAGGCTGAAGAAGATTGAGGCGCAGCTTTTGTCCAGGACATGGTTAGTTCGTTCTCTAGAAT 630
Fv149  ----------AGTTCCAGAAGACTG---------CCTCTTTCGGG------------------------- 209

Fv999  GAATAATTATATGCTAATACCAAGACAGATTCAAGACTTTCTTTACGCTCATTCCCATGTCGGGCTGGAC 700
Fv149  ------TCACATGCT----------------------------------------------------GAC 221

Fv999  CTTTGAAGAGGCGATCGTGACAAACGCCGAGGCGATGGTTGGGTGCGCTGATGGAGGTGGTGGAGAGATG 770
Fv149  CTTTGAAGAGGCGATCGTGACAAACGCCGAGGCGGTGGTTGGATGCGCTGATGGAGGTGGTGGAGAGATG 291

Fv999  TCATATGGGCGTGGTTGGAGGTGAGGGACAGTAAATTTGGGCACTGTACCCTACCTGAGTACTATGCCTG 840
Fv149  TTATATAGGCGTGGTTGGAGGTGAGGGACAGTAAATATGGGCACTGTACCCTACCTGAGTACTACGTCTG 361

Fv999  ACGCAGTATTCATATGGACATGCACTCATTCTTGTACGTCATGATAAGTCCATCCATCATATCATAGACT 910
Fv149  ACACAGTATTAATATGGACATGCACTCAGTCTTGTACGTCATGATAAGTCCATCCATCATATCAT----- 426

Fv999  ACCCCGCCATGAACTGGAGTGCTTGACTCTATCTCGGCGGCTTTAATCCCAATACGAAATTGTTATCTCG 980
Fv149  ACCCCGCCATGATCTGGAATGCTTGGCTCTATCTCGGCGGCTTTGATCCCAATACGAAAATCTTGTCTCA 496

Fv999  AGGTTTTCGTTTATCACAATCTCCATGTTACAGATCATTTATGCAAGTCATAAATCTAACATTAAATCAA 1050
Fv149  AGGTTTTCGTTTATCAGAATCCCCATGTCACAGATCATTTATGCAAGTCATAAATCTAACATGAAATCAA 566

Fv999  TTGTTTGTTGCCTATCGCTATGCGTACTCCATAATCCGTCCGAAATCGCAGCCAGTTCAATTTACAGCAG 1120
Fv149  TTGTTTGTTGCCTATCGCTATGCGTACTCCATAATCCATCCGAAATC-CAGCCCCTTCAATTTACAGCAG 635

Fv999  TCTCC---CTCTGATGGTCTCA----CCACACCCACCATCAATAATTGTCGCGTGTTTCTAAGCCCCCAA 1183
Fv149  TCTCCTCCCTCTGAAGGTGTCAGTCACCACACCCACCATCAATAATTGTTGCGTGTT-CTGAACCCCCAA 704

Fv999  TGCTAAGCCATTCGCCATGATCTGATGTTTAGTGTACAACCTTTGCATCTACTCACTTCTAGATCTCGTC 1253
Fv149  TGCTAAGCCATTCGCCATGGTCTGATGTTTAGTGTACAAACTTTGCATCTACTCACTTCTAGATCTCGTC 774

          FVEG_03164
Fv999  GTTTACTGCTTCTTCTTCTT 1273
Fv149  GTTTACTGCTTCTTCTTCTT 794
```

Figure S2 A Clustal W alignment of the *FVEG_03165-FVEG_03164* intergenic region from Fv999-*Sk*^{K} and Fv149-*Sk*^{S}. The coding start site of flanking gene *FVEG_03165* is marked with a blue arrow. Similarly, the coding stop site of flanking gene *FVEG_03164* is marked with a blue line. The predicted coding sequence of *SKC1* is marked by a red line. This alignment shows that *SKC1* is present in Fv999-*Sk*^{K} but not Fv149-*Sk*^{S}.
