# Supplementary material for: A Meiotic Drive Element in the Maize Pathogen Fusarium verticillioides Is Located Within a 102 kb Region of Chromosome V
Source: G3 (Bethesda). 2016 Jun 10;6(8):2543–52. doi: 10.1534/g3.116.029728 (PMC4978907; doi:10.1534/g3.116.029728)
Supplement: Supplemental Material [file supp_g3.116.029728_FigureS3.pdf]

```
                                  10        20        30        40        50
                         ....|....|....|....|....|....|....|....|....|....|
Fv EWG40958.1            MSSNKSSRALRTEVSKLARLSNIQGGLPPLMRGLPVLAIMAGEAIAKTTN 50
Fo EWZ36731.1            MSSNKSSQALRTEVSKLARLSNIQGGLPPLMRGLPVLAIMAGEAIAKTTN 50
Ff CCT68240.1            MSSNKSSQALRTEVSKLARLSNIQGGLPPLMRGLPVLAIMAGEAILKITN 50
Nd KPM38041.1            MPPSRTFPSLQTELSKAAKLSNVQAGLPPLIRGLPVLAILAGEVVAKATN 50
Sa XP_016641970.1       MLYSKNSVYLKSEISKLAKLGNIQGGIPPVMRGLPVLAIMAGEAVAQATH 50
Af KMK58118.1            MPGNKLPIDLAAEISKLAKASRVQSGLPGSMRALPAIALFAGTALSKTTG 50

                                  60        70        80        90       100
                         ....|....|....|....|....|....|....|....|....|....|
Fv EWG40958.1            TTNMIEERMLGADEVFILKSPQESTRSTVLKDIGRPGVKLMCQTCGQKLD 100
Fo EWZ36731.1            TTNMIEERMLGADEVFILQSPQESTRSTAMNDIGRPGVNLMCQTCGQKTG 100
Ff CCT68240.1            TTNMIEERMLGADEVFILQSPQESTTSPAKNDFGRPGVKLMCQICGQKAG 100
Nd KPM38041.1            GQNAVEERILGFDEAVLLQAPTRQLSLTETHVWARPGIAEMCQMCNKTKS 100
Sa XP_016641970.1       GRNFIEERMLGADEAILLGAPPKQLPLTTRGDAARPGGVD---------- 90
Af KMK58118.1            TSNVVEKWMLGDEEVPLLGSLTVGNPDKNVATQTGDGQCN---------- 90

                                 110       120       130       140
                         ....|....|....|....|....|....|....|....|..
Fv EWG40958.1            KIERVGVFDILRGHGSLKGRHQISRNGDS----NGGV----- 133
Fo EWZ36731.1            KIQKEAMFDISRAHNSLTGYHQISRNGGSALMPERDMETMRR 142
Ff CCT68240.1            KIQRVGVLDISRAHSSLTEYHQMSGNGNS----NSDV----- 133
Nd KPM38041.1            KSPIVTQTDVLRA----------------------------- 113
Sa XP_016641970.1       REPGTEEKP--------------------------------- 99
Af KMK58118.1            HQGRSDEQA--------------------------------- 99
```

Figure S3 Sequence alignment of putative Fveg_15999 homologs. A BLASTP search of NCBI's non-redundant protein database was performed with the sequence of *F. verticillioides* Fveg_15999. The sequences of five putative homologs were downloaded, imported into Bioedit 7.2.5, and aligned with Clustal W. Columns were shaded according to the BLOSUM62 similarity matrix. Each sequence is identified by its accession number: EWG40958.1 (Fveg_15999), *F. verticillioides*; EWZ36731.1, *F. oxysporum* Fo47; CCT68240.1, *F. fujikuroi*; KPM38041.1, *Neonectria ditissima*; XP_016641970.1, *Scedosporium apiospermum*; KMK58118.1, *Aspergillus fumigatus*.
