# Supplementary material for: A Meiotic Drive Element in the Maize Pathogen Fusarium verticillioides Is Located Within a 102 kb Region of Chromosome V
Source: G3 (Bethesda). 2016 Jun 10;6(8):2543–52. doi: 10.1534/g3.116.029728 (PMC4978907; doi:10.1534/g3.116.029728)
Supplement: Supplemental Material [file supp_g3.116.029728_FigureS4.pdf]

```
                          10        20        30        40        50
                  ....|....|....|....|....|....|....|....|....|....|
Fv EWG40990.1     MCRETITIAQCAPEVSPVLAFYCAKLHLVAKDRIICDATKGKCICFFGSC 50
Ff KLP03525.1     MCSETITVAQCAPKVSPVLAFYCAKLHLVAKERIVCEAAKGKCICFFGSC 50
Fo EWZ36701.1     MCRETITIAQCAPEVAPVLAFYCAKLHLVAKDRTVCDAARGKCICFFGSC 50
Fg ESU17197.1     MCVETITIALCAPQSAPRLSFTCGNLHLVAENHVVCDSAVGNCICYFGTC 50
Fl KPA39364.1     MCVETITIALCAPESSPHLSFTCGKLHLVAGRRTVCDKASGRCVCYFGTC 50
Nh XP_003053980.1 MCHEVITIALCAPTKAPSLSFTCGKLHMIAQQRHVCDRARGSCVCFFGTC 50
Tv XP_013952680.1 MCKEFLTIAICAPQKAPSMSFTCGQLHLVAERRQVCDKATGACLCFVGTC 50
Ac KFH42786.1     MCSEDIAVALCAPVERRALSYTCSKLHLIASNRHVCFRAKGNCVCFFGTC 50
Th KKO99655.1     MCKEYTTIAICAPQKAPSMSFTCGQLHQVVDRRQVCDKATGSCICFVGTC 50

                          60        70        80        90       100
                  ....|....|....|....|....|....|....|....|....|....|
Fv EWG40990.1     GTIEREIPTKGINLGEVVKARCAECTPREHRVRD-IPAQEILESVLLR-S 98
Ff KLP03525.1     GAIDREIPTKGINLSDIVKARCAECTPREHRVGNGIPAQQILESVLLR-S 99
Fo EWZ36701.1     GTIDREIPTKGINLGDVVKVRCAECTPREHRVGNGLKAQQILESVLLR-S 99
Fg ESU17197.1     GKVIREVPTGGIDFSAISKVRCTTCTAREDEVGDARKDREILESVLLGRP 100
Fl KPA39364.1     GNAGRAALTDGIDFSAISKVRCTSCTAREDAVGDRRKNQEVLESVLLDPS 100
Nh XP_003053980.1 GTVEKNASTSGIDLSAIEKIRCAECTTREDNVGDRRDAKQIIESPLLQRI 100
Tv XP_013952680.1 GVLESVPSTQGLRMSSVKSIRCAACTSREEEVGDRRTNEELIESPLLTRT 100
Ac KFH42786.1     GTVERTFDS-VFDLTDLPKVRCSVCTVREDDVGHSSAAREILESPVPTRK 99
Th KKO99655.1     GVLETIPSTLAIPIASLKSIRCVACTTREEEIGDRRSNEELIDSPLLTRA 100

                         110       120       130       140       150
                  ....|....|....|....|....|....|....|....|....|....|
Fv EWG40990.1     TDGQNG---WEERSKLLSELWLNKTACPYHVDTSAEP---VAPVINITES 142
Ff KLP03525.1     ADAQNG---WEERSKLVSELWLNKTACPYHVDTSAEPATPVAEVINITEH 146
Fo EWZ36701.1     VDARNG---WEERSKLISELWLNKPACPYHVETSTEPAGPITGAINITQH 146
Fg ESU17197.1     ATNDAD---WDQTSKRLQQLWQQKSACPYHVQGSVRK---AKATVNKPAK 144
Fl KPA39364.1     AVHYVD---VDRKVKLLSQLWHQKSDCPYHAEG----------------- 130
Nh XP_003053980.1 PIEPTDDFVNKKHSAMLHELWGGARTCPFHLQPDRPR------------- 137
Tv XP_013952680.1 AIKSSEQ--KIKFDEIIKDLWNGKEDCPYHVLSTVRE------------- 135
Ac KFH42786.1     AIPEEG---LERYMRTLKSMWGSMDHCPYHVTEHENQ--------EMAAH 138
Th KKO99655.1     AVKSTED--MQHFKAIIKSLWNGEDECPYHTMLTTRE------------- 135

                         160       170       180       190       200
                  ....|....|....|....|....|....|....|....|....|....|
Fv EWG40990.1     IHLDTEVGHYGTGLSAESAGPTDSTPESAL-------ELKPAFEATVGKD 185
Ff KLP03525.1     IHLDTEVGHTGTGLSAESVVPTDPASESP--------ELKPGFEAAVQKD 188
Fo EWZ36701.1     VHFDADAGRSGVGLTAELAGPTKPTHEHAFDPTTESAELKPGFEAAVEKN 196
Fg ESU17197.1     EQVQAQVDIKSAVPSAPSKASFGKVVIDKD-----------DLHQSATTD 183
Fl KPA39364.1     ----------PVIDSASAEASFDEVSIEKQ-----------GPVQSATTD 159
Nh XP_003053980.1 ---QPSASTVNVQTKRFDERPVTPQTNDES----------VTKSHSMSRD 174
Tv XP_013952680.1 -----GSNSFPVTSKNLSDMEMDKEEDKDG------------VEKSLTAS 168
Ac KFH42786.1     HQGDAETNENESEFYDQHDNGSRQPNDDGD--------VDTKPRNDNENE 180
Th KKO99655.1     -----TSEDTPVIPKLVSVADMNTNESIS--------------KEVPIAD 166

                         210       220       230       240       250
                  ....|....|....|....|....|....|....|....|....|....|
Fv EWG40990.1     VGEFSSSDNAAMKDASSSSVGTVFTEPIFTDSLVAEPASTLADEWTANAG 235
Ff KLP03525.1     FGDFSSSDNAAMKDASSSLVG-----PVFTDSFVAEPASSLVDEWT---- 229
Fo EWZ36701.1     FSGFSSSEHAALKDASSSPVE-----PIFTDSLVAEPASSPADEWT---- 237
Fg ESU17197.1     ASEHSEADSASAVDPWT-------GAPVIAEPVTAEPAGFDSPSVK---- 222
Fl KPA39364.1     VFEHNEADSASAVDAWT-------SAPVSVNTDITEIADFDSPSDK---- 198
Nh XP_003053980.1 SVDKGWTSQAATNNSSKS-------------KLSRKSSASPTGIAS---- 207
Tv XP_013952680.1 SGEDAISDNVSEASSNAT----------------HGSNGSLNSNAS---- 198
```

```
Ac KFH42786.1         TSEPPKEESPRTYDPVKG--------ELRTDDENKASETTNHNKCP---- 218
Th KKO99655.1         VHEKVADDNVSEASSNAT----------------HGSNGSLASNLS---- 196

                                260       270       280       290       300
                      ....|....|....|....|....|....|....|....|....|....|
Fv EWG40990.1         TVNTADEWNTNTAPTGEQDGNANNHASDSGAKNILDPDEVGISTSRWAPG 285
Ff KLP03525.1         ---------TNTAPGGGHGDNTENHSSDSGVKDILDPEEVGISTSRWAPG 270
Fo EWZ36701.1         ---------TNTAPTGEQRDNADNHGFDSGVKNILDPDEVGISTSRWAPG 278
Fg ESU17197.1         ---------DDVTTVIAND--------NDGGTSVKTGSDAGKGGHNGLEG 255
Fl KPA39364.1         ---------DHASIVGDGNDDVTTGFINDGDASVKAGSDTDKGVQNGLEG 239
Nh XP_003053980.1     ---------VDSAVGLGSSRWAPKNLEEAAARSVKNQSPPTQKRPSPLQK 248
Tv XP_013952680.1     ---------QESKKKDTID--LGRKVGLQASIWANAPDKSPKGIKHTSSP 237
Ac KFH42786.1         ---------QNNNVVNGDDGIEDASDEVEAASGAEDSPKTSDSASRPMTG 259
Th KKO99655.1         ---------QESSKKKNEDSGLGRKVGLQASIWANAPAKTPERKKHNGSP 237

                                310       320       330       340       350
                      ....|....|....|....|....|....|....|....|....|....|
Fv EWG40990.1         NSPT-ASKPTNPAVDFKEPESGEEHNQAQEQAQTPVVAPSRRAVNFSYNP 334
Ff KLP03525.1         NSPTNSTKPTKPAVDFKDNESGEER----EKVQTPVVAVSRRAVNFSYNP 316
Fo EWZ36701.1         NSPSNSSQPAELAFEVRDNEKGQEQ------EQTPVVAVSRRAVNFSYDP 322
Fg ESU17197.1         DVDTKLGHDAPKSID-------------------GATPVSRRAVNFAFSA 286
Fl KPA39364.1         ----------------------------------GTTLVTRRAVNFSYSA 255
Nh XP_003053980.1     QSSP----------------------------PSQKRIVPAAAPVFVADP 270
Tv XP_013952680.1     QVQS-----------------------------PVKRQKAATPQG----- 253
Ac KFH42786.1         IEASQWAPRNEAPNAD---------------LHVQAPADSTPPSPAPKAI 294
Th KKO99655.1         KSQS-----------------------------PVKRQKPVVPQGRKPAV 258

                                360       370
                      ....|....|....|....|....|.
Fv EWG40990.1         SNAEKLMETAEIFANLKKAFLMGKA- 359
Ff KLP03525.1         DNAEKLLETAEMFANVKKAFLMGKA- 341
Fo EWZ36701.1         NNAEKLLETAEKFANVK-AFLMAKA- 346
Fg ESU17197.1         EAADKLRDTTAKFASLKSSFLMGKPI 312
Fl KPA39364.1         EAADKLRDATSKFASLKSGFLMGKAT 281
Nh XP_003053980.1     GNASKVRHATKKMANMKAFLSGTPMF 296
Tv XP_013952680.1     ---PKTRMNLPQIAAAKNFLREGSA- 275
Ac KFH42786.1         AASRPPTLTAEKIASMKEFLRMN--- 317
Th KKO99655.1         ALVKKTKFNLPQIAAAKSFLKATMV- 283
```

Figure S4 Sequence alignment of putative Fveg_03194 homologs A BLASTP search of NCBI's non-redundant protein database was performed with the sequence of *F. verticillioides* Fveg_03194. The sequences of eight putative homologs were downloaded, imported into Bioedit 7.2.5, and aligned with Clustal W. Columns were shaded according to the BLOSUM62 similarity matrix. Each sequence is specified by its accession number: EWG40990.1 (Fveg_03194), *F. verticillioides*; KLP03525.1, *F. fujikuroi*; EWZ36701.1, *F. oxysporum*; ESU17197.1, *F. graminearum*; KPA39364.1, *F. langsethiae*; XP_003053980.1, *Nectria haematococca*; XP_013952680.1, *Trichoderma virens*; KFH42786.1, *Acremonium chrysogenum*; KKO99655.1, *Trichoderma harzianum*.
