# Supplementary material for: A Meiotic Drive Element in the Maize Pathogen Fusarium verticillioides Is Located Within a 102 kb Region of Chromosome V
Source: G3 (Bethesda). 2016 Jun 10;6(8):2543–52. doi: 10.1534/g3.116.029728 (PMC4978907; doi:10.1534/g3.116.029728)
Supplement: Supplemental Material [file supp_g3.116.029728_FigureS5.pdf]

```
                             10        20        30        40        50
                    ....|....|....|....|....|....|....|....|....|....|
Fv EWG40993.1       MA---------------EEKK--IPVTN---------------------- 11
Fo EWZ36697.1       MA---------------EEKK--IPVTN---------------------- 11
Ff KLO94664.1       MA---------------EEKK--IPVTN---------------------- 11
Nh XP_003053984.1   MA---------------SSQREEIPITN---------------------- 13
Ct KZL78281.1       MR---------------RPRQTSFPITKLPSSPASADFISAGPSLIDATL 35
Th KKP02497.1       MS---------------LEAHD---------------------------- 7
Nc XP_960303.1      MSDNN------TTSTKYSDTREDVPTPSLACH------------------ 26
Af KMK62774.1       MS-----------------CHQHIPR------------------------ 9
Pf KTC53377.1       MSEIR-----KAHGVSMIGAALLAPILAGCAGNNSASS------------ 33
Ap WP_026019211.1    MSFLHNFSCRRCFNRTLTAALVAAPFATLSGRQANAADK----------- 39
Se WP_058683418.1    MKILP-----LAL--------FIIPFLAGCGANNTPPQ------------ 25

                             60        70        80        90       100
                    ....|....|....|....|....|....|....|....|....|....|
Fv EWG40993.1       -------------EGMGKPLSAKNQVLTAGAAVTQEFRPVKHICAHLNAF 48
Fo EWZ36697.1       -------------EGMGKPLSVKNEVLTAGAAVTQEFRPVKHICAHLNAF 48
Ff KLO94664.1       -------------EGMGNPLSAKNQVLTAGAAVTQEFRPVKHICAHLNAF 48
Nh XP_003053984.1   -------------EGMGQPLSATNQVLTAGAAVTQDFRPVKSICAHLNAF 50
Ct KZL78281.1       QSRLRPTMAAKPGSNAGEPISTKNQVLQAGASAIQDFAPLRNVCAHLNAF 85
Th KKP02497.1       -------------KAPGDLLPKEDTVLSSAASFTQDFKPVKNICAHLNAF 44
Nc XP_960303.1      --------DPSNPSIPGSPKTTLSSALETGASLAQDFSPVKSICAHLNAF 68
Af KMK62774.1       ----------DQDGIPGSPLSTKSRVLEGGASMVQDFTPVKQICAHLNAF 49
Pf KTC53377.1       ------------VVAPGAPQSPATQSLDAGAALLQSRPPIDALNAYLDGF 71
Ap WP_026019211.1    ----------KLMPVSGSSKSMRSHIIDTAADTLQTKKPIDAMSEYLNGF 79
Se WP_058683418.1    ------------TPIPGEKTSAKLRTLETGAAAIQSRPPVDAISTYLNGF 63

                            110       120       130       140       150
                    ....|....|....|....|....|....|....|....|....|....|
Fv EWG40993.1       HAYADDPSRFVETNHYCAHLN---------------EDVRQCLLYDSDEP 83
Fo EWZ36697.1       HAYADDPSRFVETNHYCAHLN---------------EDVRQCLLYDSDEP 83
Ff KLO94664.1       HAYADDPSRFVETNHYCAHLSTSLTNRSTRSLTPADEDVRQCLLYDSDEP 98
Nh XP_003053984.1   HAYADDPKRFVEANHYCAHLN---------------EDVRQCLLYDSDEP 85
Ct KZL78281.1       HAYASDPSRVVEANHYCGHLN---------------EDVRQCILYDSPEP 120
Th KKP02497.1       HAYANDPMRSVEANHYCSQLD---------------DEVRQCVLYDSPEP 79
Nc XP_960303.1      HVYASDPTRFVEANHYCAHLT---------------EDVRQCILYDSPNP 103
Af KMK62774.1       HVYASDQTRAVEANHYCTHVT---------------EDLRQCLIYDSTKP 84
Pf KTC53377.1       HFYNGHMDAQMEAHHYCAILN---------------EEVIQCVIYDGNRK 106
Ap WP_026019211.1    HFYADDMGRQIEANHFCTHLT---------------EDFHQCVIYEANQP 114
Se WP_058683418.1    HFYSGDKNGQMEAHHYVTVLN---------------EDVMQAVIYDGNTK 98

                            160       170       180       190       200
                    ....|....|....|....|....|....|....|....|....|....|
Fv EWG40993.1       NARLIGIEYMITPKLYETLDKEERKLWHSHVYEVKSGMLIMP--NRAVPE 131
Fo EWZ36697.1       NARLIGIEYMVTPKLYETLDKEERKLWHSHVYEVKSGMLIMP--NRAVPE 131
Ff KLO94664.1       NARLIGIEYMITPKLYETLDKEERKLWHSHVYEVKSGMLIMP--NRAVPE 146
Nh XP_003053984.1   TARLIGIEYMITPKLYDTLSKEERKLWHSHAYEVKSGMLIMP--NRAVPE 133
Ct KZL78281.1       NARIIGIEYMITPKLYETLDAEERKLWHSHVYEVKSGMLIMP--QMTLPD 168
Th KKP02497.1       NARIIGIEYMITPKRYESLPADERRLWHSHVYEVKSGMLVMP--NRMVPK 127
Nc XP_960303.1      GARILGIEYMITPKLYETLPQQERRYWHTHVFEVKSGMLIMPKPPGLMPQ 153
Af KMK62774.1       NARLIGVEYMISPRLFETLPSEERKLWHTHEFEVKSGMLIMPAPAG-VPK 133
Pf KTC53377.1       DAKLMGVEYIISEQLFAGLPKSEQALWHSHVHEVKSGQLVAP-------- 148
Ap WP_026019211.1    NARLIGIEYIVSERIFKTLPDDEKKLWHSHHYEVKSGMLVAP-------- 156
Se WP_058683418.1    NARLMGVEYIISERLFKTLPPEEKKLWHSHQYEVKSGSLVAP-------- 140

                            210       220       230       240       250
```

```
                      ....|....|....|....|....|....|....|....|....|....|
Fv EWG40993.1         SAWQVAENYEMDQVVHLYGKVYHLWQTDRGDTLPLGEPKLMTSFTADG--  179
Fo EWZ36697.1         SAWQVAENYEMDQVVQLYGKVYHLWQTDRGDTLPLGEPKLMTSFTADG--  179
Ff KLO94664.1         SAWQVAENYEMNQVVQLYGKVYHLWQTDRGDTLPLGEPKLMTSFTADG--  194
Nh XP_003053984.1     SAWEVAENMEMDQVVHLYGKVYHLWQTDRGDMLPLGPPKLMTSFTADG--  181
Ct KZL78281.1         AVWETAENKEMEQVVHLYGKVFHLWQVDKGHQLPLGEPQLMTSFTQPG--  216
Th KKP02497.1         AAWELAEKREMEKIITLYGKAYHLWQTDRGDELPLGEPQLMMSYTSDG--  175
Nc XP_960303.1        AAWEKAEKTEMEKVVTLYGKIYHLWQVDRGDKLPLGEPQLMTSFTDES--  201
Af KMK62774.1         PAWEAAETSEMREVILLYGKTYHFWQADRGDPLPLGAPQLMASFTSEDKV  183
Pf KTC53377.1         GIPEIAEHALMEKLVHTYGKTWHTWHTDLNKDLPLGVPQLMMGFTADG--  196
Ap WP_026019211.1     GVPNPGEHAFMTDLINTYGKTWHTWQIDRDHDLPLGIPQLMMGFTKDG--  204
Se WP_058683418.1     GLPQVADKALMSKIVNTYGKTWHTWHTDRDKTLPMGIPALMMGFTGDG--  188

                                260       270       280       290       300
                      ....|....|....|....|....|....|....|....|....|....|
Fv EWG40993.1         ----QFDFEKHVGERDRKF-GTDWRVKKEARKDIPSLVVHEDADQAWKNK  224
Fo EWZ36697.1         ----QFDFEKNVGERDRKF-GTDWRVKKEARKNIPSPVVHEDADQAWKNK  224
Ff KLO94664.1         ----QFDFEKHVGERDRKF-GTDWRVKKEARKDIPSPAVHEG-EYEWVDE  238
Nh XP_003053984.1     ----QLDFK-HVRDRDRRF-GVDHKQKREARKDIPVPAIHEDADQAWKNQ  225
Ct KZL78281.1         ----QFDFEKYVGERDRRF-KSDYKRKVEARKHIESPSVHPDADQAWKNH  261
Th KKP02497.1         ----HLDFG-RVEERDERF-GTNYREKREARKDIPSPKIHGDADSCWHAW  219
Nc XP_960303.1        --QFPVDMEKVLDERDQRFPGADWRAKREGRKDIEVPEMHPDADWTWKKD  249
Af KMK62774.1         KLVHPKGLDGLLAGRDEAF-GVDYKVKAEKRKDIEPPNLSPDADTMV---  229
Pf KTC53377.1         ----QADPQ-MIAERDRRF-GIESAQKKKARADIPAPTVAPGADAWKQGK  240
Ap WP_026019211.1     ----QIQKA-SLDDRDKRF-GISTQSERENRADIPMPDVVMGANSWQSGK  248
Se WP_058683418.1     ----QLDLA-LLADRDRRL-GIDTRAIKRERQDLPEHPVVKGANAWEQGE  232

                                310       320
                      ....|....|....|....|....|....
Fv EWG40993.1         -----------------------------  224
Fo EWZ36697.1         -----------------------------  224
Ff KLO94664.1         GLR--------------------------  241
Nh XP_003053984.1     -----------------------------  225
Ct KZL78281.1         NKEA-------------------------  265
Th KKP02497.1         RERGNKTP-------------PSYNQK--  233
Nc XP_960303.1        KQK--------------------------  252
Af KMK62774.1         -----------------------------  229
Pf KTC53377.1         VFQIE-----------DPTH--TPHQH--  254
Ap WP_026019211.1     TMQLQ------------LVEVPVKNLKK-  264
Se WP_058683418.1     VIQLQRVQGSGEHGRGDTAHFGTSEQSRQ  261
```

Figure S5 Sequence alignment of putative Fveg_03197 homologs A BLASTP search of NCBI's non-redundant protein database was performed with the sequence of *F. verticillioides* Fveg_03197. The sequences of ten putative homologs were downloaded, imported into Bioedit 7.2.5, and aligned with Clustal W. Columns were shaded according to the BLOSUM62 similarity matrix. Each sequence is specified by its accession number: EWG40993.1 (Fveg_03197), *F. verticillioides*; EWZ36697.1, *F. oxysporum*; KLO94664.1, *F. fujikuroi*; XP_003053984.1, *Nectria haematococca*; KZL78281.1, *Colletotrichum tofieldiae*; KKP02497.1, *Trichoderma harzianum*; XP_960303.1, *Neurospora crassa*; KMK62774.1, *Aspergillus fumigatus*; KTC53377.1, *Pseudomonas fluorescens*; WP_026019211.1, *Acetobacter pasteurianus*; WP_058683418.1, *Salmonella enterica*.
