# Supplementary material for: A Meiotic Drive Element in the Maize Pathogen Fusarium verticillioides Is Located Within a 102 kb Region of Chromosome V
Source: G3 (Bethesda). 2016 Jun 10;6(8):2543–52. doi: 10.1534/g3.116.029728 (PMC4978907; doi:10.1534/g3.116.029728)
Supplement: Supplemental Material [file supp_g3.116.029728_TableS1.pdf]

**Table S1 Oligonucleotide primers for CAPS markers**

| Name | Sequence (5’ to 3’) |
|---|---|
| CAPS-1F | GCGATACAGAACCCCCATTCTCTT |
| CAPS-1R | GATCAGCCATCCTATCTCTCCCAGT |
| CAPS-2F | TTATCTGCACACCTGGAGGA |
| CAPS-2R | CGGCTTCACCTGAGACATTT |
| CAPS-3F | CCAGAATGGCTCTGACCTGGTGT |
| CAPS-3R | GCGCAGATCATGGAGAGGGTATG |
| CAPS-4F | ATGTGTGCAGCTCGTTTTTG |
| CAPS-4R | TCAACCCCGAGACTTTCATC |
| CAPS-5F | GACCAGACCCGAAACCAAT |
| CAPS-5R | CAGCCAAATCACGCTGTCT |
| CAPS-6F | GGTCGAGAACAAAGGGGTTC |
| CAPS-6R | CAAAGATGGGAGGAACATGG |
| CAPS-9F | CGCGGTTGACACGGCTCTC |
| CAPS-9R | GAAATGCGGCTCCAACTTCTCG |
| CAPS-10F | TGCTGCCTCGATTCCTTCTTCC |
| CAPS-10R | CGGAGTACCATTGTTCGGGTGA |
| CAPS-11F | TGCACAGAAGCGAGACAAACATCC |
| CAPS-11R | TCGAGCATGACCAAGGCAGAAC |
