# Supplementary material for: A Meiotic Drive Element in the Maize Pathogen Fusarium verticillioides Is Located Within a 102 kb Region of Chromosome V
Source: G3 (Bethesda). 2016 Jun 10;6(8):2543–52. doi: 10.1534/g3.116.029728 (PMC4978907; doi:10.1534/g3.116.029728)
Supplement: Supplemental Material [file supp_g3.116.029728_TableS2.pdf]

Table S2 CAPS marker sizes

marker	PCR product length	Fv149 product lengths after *Hae*III digest	Fv999 product lengths after *Hae*III digest
CAPS-1	358	44, 86, 228	151, 207
CAPS-2	307	132, 175	307
CAPS-3	311	311	105, 206
CAPS-4	288	110, 178	288
CAPS-5	265	119, 146	265
CAPS-6	325	34, 92, 199	34, 291
CAPS-9	291	128, 163	291
CAPS-10	404	404	68, 336
CAPS-11	333	333	108, 225
