# Supplementary material for: A Meiotic Drive Element in the Maize Pathogen Fusarium verticillioides Is Located Within a 102 kb Region of Chromosome V
Source: G3 (Bethesda). 2016 Jun 10;6(8):2543–52. doi: 10.1534/g3.116.029728 (PMC4978907; doi:10.1534/g3.116.029728)
Supplement: Supplemental Material [file supp_g3.116.029728_TableS3.pdf]

Table S3. Genotypes of the Fv999 (*SK*^{K}) × Fv149 (*SK*^{S}) mapping population at each CAPS location

Strain	CAPS-5	CAPS-4	CAPS-3	CAPS-2	CAPS-1	CAPS-6	CAPS-9	CAPS-10	CAPS-11
#60	S	K	K	K	K	K	K	K	K
#61	S	S	K	K	K	K	S	S	nd
#62	K	K	K	K	K	K	S	K	S
#63	K	K	K	K	K	K	K	K	S
#64	K	K	K	K	K	K	S	K	K
#65	K	K	K	K	K	K	S	K	S
#66	K	K	K	K	K	K	K	K	K
#67	S	K	K	K	K	K	S	S	S
#68	K	K	K	K	K	K	S	S	nd
#69	S	K	K	K	K	K	S	K	K
#70	S	K	K	K	K	K	nd	S	S
#71	S	S	K	K	K	K	nd	K	K
#72	K	K	K	K	K	K	K	S	S
#73	K	K	K	K	K	S	S	S	S
#74	K	K	K	K	K	K	K	K	K
#75	S	S	K	K	K	K	K	S	S
#76	S	K	K	K	K	K	K	S	S
#77	S	K	K	K	K	K	S	K	S
#78	K	K	K	K	K	K	S	K	K
#79	S	K	K	K	K	K	S	K	K
#80	S	S	K	K	K	K	S	K	K
#81	K	K	K	K	K	K	K	K	S
#82	S	K	K	K	K	K	K	K	K
#83	K	K	K	K	K	K	K	K	K
#84	S	K	K	K	K	K	K	K	S
#85	K	K	K	K	K	K	K	S	K
#86	K	K	K	K	K	K	K	K	K
#87	S	S	K	K	K	K	K	K	K
#88	S	nd	K	K	K	K	K	S	S
#89	S	K	K	K	K	K	K	S	K
#90	S	K	K	K	K	K	K	K	S

#91	K	K	K	K	K	K	K	K	K
#92	S	K	K	K	K	K	K	nd	K
#93	S	K	K	K	K	K	K	K	K
#94	S	K	K	K	K	S	K	K	S
#95	K	K	K	K	K	K	K	K	S
#96	K	K	K	K	K	K	S	K	S
#97	K	K	K	K	K	S	K	K	S
#98	K	K	K	nd	K	K	K	K	K
#99	K	K	K	K	K	K	K	S	S
#100	S	K	K	K	K	K	K	K	S
#101	S	S	K	K	K	K	K	S	K
#102	K	K	nd	K	K	K	S	K	K
#103	K	K	K	K	K	K	S	K	K
#104	S	K	K	K	K	S	S	S	S
#105	S	K	K	K	K	S	K	K	S
#106	S	S	K	K	K	S	S	S	S
#107	K	K	S	K	K	K	S	S	S
#108	K	S	K	K	K	K	K	nd	K
#109	K	K	K	K	K	K	K	K	S
#110	S	S	K	K	K	S	S	S	K
#111	K	S	S	S	S	S	K	S	K
#112	K	K	K	K	K	K	S	nd	K
#113	K	K	K	K	K	K	K	S	S
#114	K	K	K	K	K	K	K	S	S
#115	K	K	K	K	K	nd	S	S	K
#116	S	S	K	K	K	K	K	K	K
#117	K	K	K	K	K	K	K	S	K
#118	K	K	K	S	S	S	K	K	K
#119	S	S	K	K	K	K	K	S	K

The chromosome V CAPS markers (1 through 6) are arranged in the table according to their relative order on the chromosome. K = Fv999-Sk^K genotype; S = Fv149-Sk^S genotype; nd = not determined.
